# Circumcision does not alter long-term glucocorticoids accumulation or psychological effects associated with trauma- and stressor-related disorders

**DOI:** 10.1038/tp.2017.23

**Published:** 2017-03-14

**Authors:** E Ullmann, J Licinio, A Barthel, K Petrowski, B Oratovski, T Stalder, C Kirschbaum, S R Bornstein

**Affiliations:** 1 Department of Medicine, TU Dresden, Carl Gustav Carus, Dresden, Germany; 2 Department for Child and Adolescent Psychiatry, Psychotherapy, and Psychosomatics, University of Leipzig, Leipzig, Germany; 3 Mind and Brain Theme, South Australian Health and Medical Research Institute, Flinders University, Adelaide, SA, Australia; 4 Endokrinologikum RUHR, Bochum, Germany; 5 German Sport University, Cologne, Germany; 6 Department of Psychology, TU Dresden, Dresden, Germany; 7 Clinical Psychology, University of Siegen, Siegen, Germany; 8 Faculty of Life Sciences & Medicine, Endocrinology and Diabetes, Kings College London, London, UK

## Abstract

Male infants and boys through early adolescence can undergo circumcision either for the sake of upholding religious traditions or for medical reasons. According to both, Jewish as well as Islamic tenets, circumcision is a religious rite symbolizing the bond with God. The World Health Organization (WHO), the United Nations Council (UNC) as well as the American Academy of Pediatrics (AAP), and the Centers for Disease Control and Prevention (CDC) strongly recommend circumcision to promote hygiene and prevent disease. This procedure has frequently been criticized by various communities claiming that circumcision in infancy and early adolescence were psychologically traumatizing with medical implications up into old age. Due to the lack of evidence concerning an alleged increase in vulnerability, we measured objective and subjective stress and trauma markers, including glucocorticoids from hair samples, in circumcised and non-circumcised males. We found no differences in long-term limbic–hypothalamic–pituitary–adrenal axis activity, subjective stress perception, anxiety, depressiveness, physical complaints, sense of coherence and resilience. Rather, an increase in the glucocorticoid levels indicated a healthy lifestyle and appropriate functioning. Thus, our findings provide evidence that male circumcision does not promote psychological trauma. Moreover, a qualitative approach, the ambivalence construct, was used for the discussion, aiming at a discourse devoid of biases.

## Introduction

Circumcision is a religious and medical procedure for infants and males in early adolescence, whose long-term effects are unresolved. Many worldwide refugees, in part including severely traumatized children, are of Abrahamic origin.^1^ The sons of believers from these religious communities undergo circumcision as a sign of the bond with God as described/prescribed in the *Talmud* (*Genesis 17:10*) *and the Qur'an* (*Sure 16, 123*). From a medical perspective, circumcision of male infants shortly after birth is recommended as the long-term health benefits of newborn male infant circumcision is said to outweigh any risks.^2^ Moreover, the World Health Organization (WHO), the Centers for Disease Control and Prevention (CDC) and the United Nations Council (UNC) recommend medical male circumcision as a key intervention in generalized epidemic settings, for example with high HIV prevalence and low circumcision rates.^3, 4^ Since these recommendations were announced, many intense, emotionalized discussions have ensued in order to balance medically indicated male circumcision with cultural influences.^5, 6^

Some argue that circumcision has a mentally traumatizing effect on male infants resulting in autism spectrum disorder (ASD) or the hyperkinetic disorder.^7, 8^ Reliable data regarding the development of trauma- and stressor-related disorders (TSRDs) as a result of male circumcision are rather limited. Circumcision status was not associated with health in infancy, cognitive ability outcomes in later childhood or breastfeeding.^9^ On the other hand, reports usually include subjective descriptions by adult observers based on the visual appraisal of neonatal suffering.^10^ Quantitative reports indicate higher intravenous cortisol levels in neonates ~30 min following circumcision conducted 57–80 h after birth.^11^ Decreased cortisol levels from an enhanced limbic–hypothalamic–pituitary–adrenal (LHPA) axis feedback are a neurobiological marker for higher vulnerability in patients with a posttraumatic stress disorder (PTSD) and depression.^12^ Recently, alterations within the LHPA axis have occurred following psychological trauma in early childhood due to a decrease in the efficiency of negative feedback of the stress hormone axis as traced through old age with epigenetic markers.^13^ As a way of proving the functionality of the LHPA axis, via hair glucocorticoid measurements we were able to demonstrate that a long-term LHPA axis activation occurs after physical activity but also after mental hardships and PTSD.^14, 15, 16^ A reduction in cortisol concentrations accompanies a depletion of the LHPA axis, indicating an overload or exhaustion of the LHPA axis as has been observed in descendants of Holocaust survivors.^17, 18^

We hypothesized, if circumcision were mentally traumatizing, circumcised men would show a decreased long-term activation of the LHPA axis as assayed in hair from physical activity due to the lower efficiency of negative feedback mechanisms. We also presumed that circumcised Jewish men in the third generation following the Holocaust would show lower long-term glucocorticoid concentrations in hair than the comparative group of uncircumcised Jewish men in the third generation following the Holocaust.

Furthermore, an increase in depressiveness and anxiety as well as in psychosomatic symptoms might thus be an indication for this kind of disorders in the aftermath of traumatic events and experiences, up to a PTSD.^19, 20^ Moreover, Jewish descendants of the third generation following the Holocaust who had immigrated to Germany from the former Soviet Union show increased depressiveness and psychosomatic symptoms.^21^ Thus, if male circumcision were a mentally traumatizing experience, circumcised Jewish males would display more anxiety, depressiveness and physical complaints than a control group of uncircumcised Jewish males without stress-related transgenerational side effects after World War II and emigration.

To develop long-term indications following psychological trauma, the moderating capabilities in afflicted individuals are paramount. Here the focus lies on personal traits such as the sense of coherence (SOC), which is an essential coping resource that allows an individual to become more resistant to stress, and resilience, which may be understood as a counterpart to vulnerability.^22, 23, 24, 25^ Accordingly, we predicted that circumcised Jewish males would possess fewer capabilities for social embedding (SOC) and psychological resilience than the control group of uncircumcised males.

The data presented in this study investigated male circumcision to reveal new insights into the pathogenesis of TSRD. Moreover, these data may present evidence concerning the long-term consequences of male circumcision. As such, these data may clarify the issues of the still ongoing emotional discussions in the natural sciences, religious studies and legal discourses.

## Materials and methods

Our cross-sectional study was approved by the Institutional Review Board of the medical faculty Carl Gustav Carus at the Technical University (TU) Dresden, whereby the study was conducted in accordance with the guidelines approved by the institutional review board. The investigation took place in the framework of a seminar by the German Central Jewish Council in September 2014. Chosen for the investigation were exclusively those seminar participants from Jewish congregations who had immigrated to Germany from the former Soviet Union. The chosen participants gave signed informed consent according to the description of the study. Due to the conditions of the standardized questionnaires used, 20 subjects were included (11 uncircumcised, 3 circumcised without analgesia, 6 circumcised with analgesia). All were older than 18 years of age. Also, we avoided post-war psychosocial transgenerational transmission influences as well as acculturation effects by including only subjects with the same immigration background, thus excluding subjects whose parents were born before 8 May 1945 and grandparents born after this date since these factors may also be related to stress. Individuals known to suffer from Cushing's disease, Addison's disease, hypo-/hyperthyroidism or other endocrine disorders were excluded for the same reason. Then, questionnaires were filled out by the subjects, and hair samples were taken.

### Socio-demographic and hair-related characteristics

The subjects were characterized with regard to the following criteria: socio-demographic, vital (height, weight) and hair-related characteristics (frequency of hair washing and hair coloring/tinting), physical activity, subjective stress perception, anxiety, depressiveness, psychosomatic symptoms, SOC and resilience. In addition, we inquired about the subjects' circumcision status and the respective date of the procedure.

Hair strands were taken scalp-near from a posterior vertex region. Glucocorticoid hormone concentrations were determined in the proximal 3 cm long hair segment which, based on an approximate hair growth rate of 1 cm per month,^26^ is assumed to reflect the integrated hormone secretion over the 3-month-period prior to hair sampling. The concentrations of cortisol and cortisone were determined by liquid chromatography–tandem mass spectrometry (LC–MS/MS), the current gold standard approach for hair glucocorticoid analysis,^27^ according to our published protocol with 7.5 mg of whole, non-pulverized hair used for the current analyses.^28^

### Questionnaires

Stress perception was recorded using the 30-item Perceived Stress Questionnaire (PSQ).^29^ The PSQ allows for the quantitation of subjective perception, evaluation and further processing of stressors by using scaled questions such as ‘you find yourself *in situations* of conflict' or ‘your problems seem to be piling up'. Also, dominance and external stressors experienced during the previous 4 weeks were quantified. Subjective stress perception is one of the most decisive factors in the course of various disorders and clinical pictures. Therefore, the information about stress perception is an essential prerequisite for improving therapeutic strategies. The PSQ meets the highest national and international quality standards (Cronbach's alpha >0.85).^29, 30^

The short form of the Giessen Complaint List (GBB-24) is a tool for the detection of psychosomatic or additional conditionality of physical complaints. The GBB-24 is used in order to differentiate between physical symptoms and subjective complaints. An aggregate value makes it possible to determine the overall burden of physical complaints. The Giessen complaint list shows excellent psychometric values (Cronbach's alpha=0.93).^31^

Anxiety and depressiveness were recorded using the Hospital Anxiety and Depression Scale (HADS).^32^ Fourteen items are specified in this scale, and an anxiety as well as a depression subscale is established. Higher scores indicate higher levels of anxiety or depressiveness. Current psychometric data validate the quality of this scale on the national as well as on the international level (Cronbach's alpha >0.80).^33, 34, 35, 36^

The subjects' SOC was investigated by the SOC Scale in its abbreviated form of nine items (SOC-9L).^37, 38^ Within the framework of Antonovsky's saluto-genetic model, the SOC plays an important role as a personal trait for the health-promotive dealing with stressors, including the activation of generalized resistance resources.^39^ The SOC is described as an outlasting element in the life span and socio-cultural context. Using the SOC, a general life orientation in the dimensions ‘comprehensibility', ‘manageability' and ‘meaningfulness' are recorded at different degrees of variation. Both, the SOC as well as its abbreviated form (SOC-9L) have proved to be solid instruments (Cronbach's alpha >0.87).^37, 38^

In addition, the abbreviated German version of the resilience scale (RS-13) by Wagnild and Young^40^ was utilized. In this questionnaire, resilience is conceptualized as the ability to use internal and external resources for coping with developmental tasks. The scale ‘Personal Competence' assesses self-value, independence, containment and persistence. The dimension Acceptance of Self and Life incorporates adaptability, tolerance and flexibility. Both the resilience scale as well as the short scale of 13 items (RS-13) are widely accepted as dependable instruments for recording psychological resilience.^40, 41^

### Further self-developed questions

The individual questions are worded as follows: how much sport are you doing per week? How actively have you been engaged in sports (1=very little to 10=very intensive)? The individual questions have already been utilized; however, they have not been published or standardized yet.

After data entry into IBM SPSS Statistics 23, we checked for statistical outliers and standard deviations (Kolmogorov–Smirnov). As a result, the data of one uncircumcised subject was excluded from analysis due to a high hair-cortisone concentration. Finally, we performed two-tailed *t*-tests, linear regression and Pearson's correlations.

## Results

The socio-demographic as well as some circumcision- and hair-related characteristics are shown in Table 1. Concentrations of glucocorticoids in hair samples were: cortisol 6.6±0.9 s.e. pg mg^−1^ (*N*=20); cortisone 16±2.05 s.e. pg mg^−1^ (*N*=19). One statistical outlier with high for hair-cortisone level was excluded in uncircumcised subjects. Physical activity (sports frequency per week) was 2.7±1.3 s.d. (*N*=15) and sports intensity was 5.6±2.1 s.d. (*N*=19).

**Figure 1:**
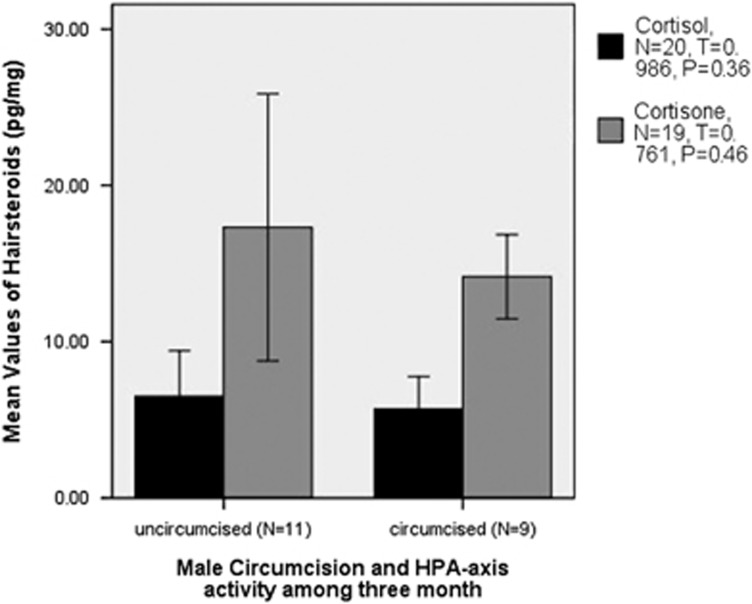
Comparisons between cortisol- and cortisone levels in circumcised and uncircumcised Jewish male subjects.

There was no significant difference in physical activity, depressiveness, anxiety, psychosomatic symptoms, SOC, resilience (Table 2) or glucorticoids (Figure 1) between circumcised and uncircumcised subjects. In uncircumcised subjects, concentration of cortisol was 7.4±1.4 s.e. pg mg^−1^ (*N*=11) and cortisone 17.3±3.8 s.e. pg mg^−1^ (*N*=10), whereas in circumcised subjects concentrations of cortisol was 5.7±0.9 s.e. pg mg^−1^ (*N*=9) and cortisone 14.2±1.2 s.e. pg mg^−1^ (*N*=9). Even when the statistically calculated outliers with increased cortisone level were included, no significant differences were found.

**Figure 2:**
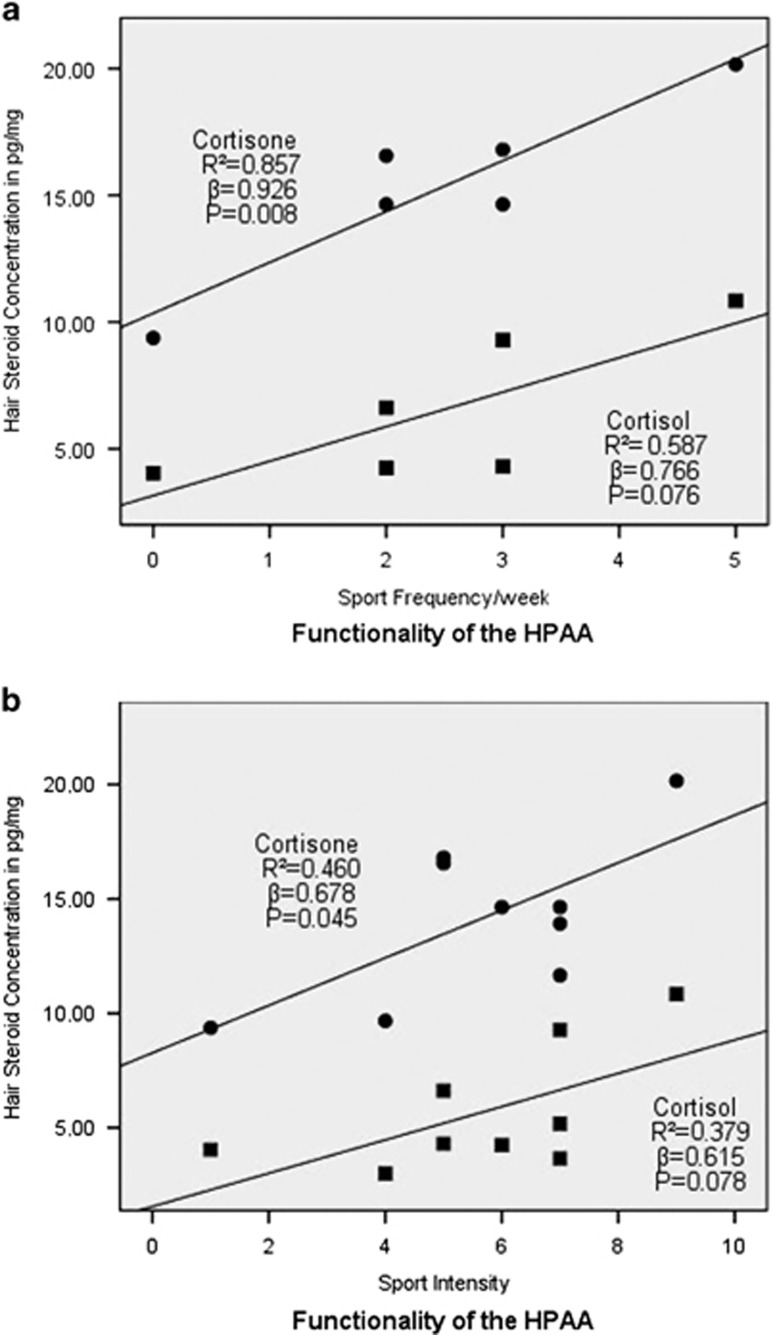
(**a**) Multiple linear regression between cortisone- and cortisol hair concentrations and sport frequency per week in last 3 months. (**b**) Multiple linear regression between cortisone- and cortisol hair concentrations and sport intensity in last 3 months.

Furthermore, we found no decreased functionality of the LHPA axis in circumcised subjects by calculating a multiple linear regression (Figure 2) concerning the concentrations of our glucocorticoids and physical activity (sport frequency and sport intensity per week).

Lastly, we verified the expected Pearson's correlations to test the reliability and integrity of the total data of the standardized questionnaires. As a result, all known correlations concerning depressiveness, anxiety, psychosomatic symptoms as well as SOC, resilience and subjective stress perception were found (Supplementary Data).

## Discussion

The data presented here compare the stress and illness burden as well as moderating cognitive capabilities of circumcised and uncircumcised males of Jewish origin. We found no statistically significant relationships in glucocorticoid hormone levels and self-report measures in our population. Our results add to the growing body of evidence in the literature that male circumcision is not likely psychologically traumatizing across the life-span. Patients with PTSD can exhibit increased anxiety, depressiveness, psychosomatic symptoms and LHPA axis alterations for several years after the traumatizing event. These changes can also be found over several generations.^1, 13, 16, 17, 18, 20, 42^ Especially the epigenetic findings with a decreased phenotyping expression of the glucocorticoid receptor after early childhood psychological trauma and the influences of the LHPA axis activity across the lifespan support our results.^13, 18^ Moreover, we had predicted that a relationship to SOC and resilience might be found if circumcision were a trauma- and stress-related issue like PTSD.^22, 23, 24, 25^ Our data however suggest that this is not the case.

Although the small sample size in our study is a limitation, the analysis concerning the functionality of the LHPA axis however supports our conclusions. On the basis of this functional test, we were able to establish that our reported physical activity patterns in circumcised Jewish males three generations following the Holocaust were the same as those in healthy subjects and athletes of the general German populace.^14, 16^ The increased cortisol concentrations in male infants following circumcision found in the literature may be from confounding variables such as the ultra- and circadian rhythms of the LHPA axis and intravenously taken blood samples without pain-relieving practices.^11^ The protocols for observation by adults witnessing newborn infant circumcision do not meet the standards of present-day scientific methods and ought to be interpreted with caution.^10^ And the diagnostic criteria for ASD or the hyperkinetic disorder applied in the Denmark cohort study is unclear since there are no information concerning the age of the subjects in the diagnostic process.^8^ By using current diagnostic criteria for ASD, a higher prevalence was reported in uncircumcised Danish boys than in circumcised (7.2% vs 6.3%).^8, 43^ Furthermore, the pre- and perinatal paracetamol/acetaminophen exposure is unclear in the Denmark cohort study that could be a plausible explanation for higher prevalence of ASD in circumcised subjects.^44^ And, there were no associations between circumcision and health in infancy, cognitive ability outcomes in later childhood or breastfeeding.^9^

Some experts criticize the cultural biases that may accompany the recommendations for the medically indicated circumcision of male infants in the United States and the American Academy of Pediatrics (AAP) argued that the cultural bias resides in Europe, not in the United States.^5, 6^ Oddly, there is no criticism to date by the experts regarding the non-existent recommendation for the regular use of pain-relieving practices before neonatal venipuncture, although there is sufficient data available for this recommendation.^45, 46^

Perhaps, this lack of interest concerning the pain-relieving possibilities in clinical routine can be interpreted in the sense of an emotional bias. The question remains why, on the one hand, the pain stimulus caused by male circumcision is so strongly objected to by some pediatricians, whereas, on the other hand, these very same pediatricians tolerate the pain stimulus caused by venipuncture. A possible explanation attempt may be Bleuler's ‘construct of ambivalence'.^47^ One may well speak of ambivalence when humans, while seeking the meaning of individuals, social contacts, and facts that are important to certain facets of their own identity and, hence, their ability to act, oscillate between the polar contradictions of feeling, thinking, wanting and social structures that seem unresolvable for a time, or even permanently. In this context, personal influence, power and domination may be relevant.

The emotionalized discussions concerning male infant circumcision are an indication of ambivalent experiences and their related sensitivities as well as vacillation dynamics and their relevance regarding the identity of the individuals involved. A holistic perspective of the ambivalence construct in a multidisciplinary sphere is needed, including the social, psychiatric, religious and parenting sciences, to come to a better understanding of how ambivalence, socialization, identity and, moreover, the different perceptions regarding the experience of pain from circumcision relate. For this, a four-dimensional heuristic approach might prove to be a suitable tool to ensure more objectivity and fewer emotionalized biases:^48^

1. ‘differentiating' between the duality counterparts,
2. ‘vacillating' in a dynamic process between the two counterparts,
3. ‘signifying' dealings with relationships and objects,
4. ‘practicing' the above-mentioned dimensions effectively.

Rituals such as circumcision are leading parts of socialization over the whole lifespan. They can reinforce identity and social embedding, but also initiate discussions. Moreover, rituals both restrict and extend the individuals, and communities that follow them. Consequently, rituals are important points of ambivalence and need to be discussed accordingly, especially in natural sciences. However, it need not necessarily be evaluated negatively, rather, it might create impulses for the birth of new ideas and research approaches. The concept of ambivalence offers an opportunity for bridging the gap between faith and rationality, or between ‘nature and nurture'.

Desirably, applying the construct of ambivalence will give rise to some interesting scientific questions. For example, how might the time that elapses between birth and circumcision itself alter the parameters adhered to in the present study? Maybe the circumcision should be done before the onset of mini-puberty of infancy (<4 weeks) or maybe the pain scores are much lower during the first week of life.^49, 50^ What is more, the circumcision practices of other countries should also be given some consideration. Investigations into Buddhist, Hindu, Islamic, Christian and Jewish societies may eventually lead to a common denominator of which moment in the life of a male individual might be the most desirable for circumcision.^51^ Perspectively, it would need to be taken into consideration that the sensitivity to pain is flanked by stress hormone axis alterations from, for example, the effects of nursing.^52^ Another, preferably cross-cultural study between circumcised US citizens and uncircumcised EU citizens might focus on stress resistance and physical capability, since an assumed LHPA axis overload caused by the possible circumcision trauma may have led to increased stress resistance due to the reduced hormone resumption at the glucocorticoid receptor.^13^ And a suitable method to reduce pre- and post-operative pain of circumcision at all ages should be investigated. In any case, it may be stated that we believe our study with Jewish male subjects has refuted the psycho-pathological long-term effects of circumcision for the first time.

## Conclusions

Male circumcision in Jewish individuals shows no changes in neurobiological (glucocorticoids) and psychometric (depressiveness, anxiety, physical complaints, resilience and SOC) markers of TSRD. A healthy functionality of the LHPA axis has been proven. In regard to clinical implications, the present study shows that measuring physical activity in association with glucocorticoid hormones in hair concentrations provides a suitable method for the determination of stress-related activity in the LHPA axis. Furthermore, persistent glucocorticoid hormone concentrations in hair samples are a cost-effective and non-invasive method. Recording the long-term hair glucocorticoid concentrations should be routine in TSRD diagnostics. Furthermore, the construct of ambivalence should be used to accomplish more empathy and mutual understanding among the opposing parties in the discussion.

One limiting factor of our study may have been the small number of subjects as mentioned above. Therefore some prudence is called for when interpreting the current results. Also, no trauma-specific methodology was used, which may have provided differential diagnostic statements to permit a more precise diagnosis of our analyses. Furthermore, the illness burden, LHPA axis alterations and moderating capabilities could only be compared several decades after the circumcision had taken place as all the circumcised subjects had been circumcised on or after the eighth day following birth.

## Figures and Tables

**Table 1:** Sociodemographic, hair-, stress- and circumcision-related characteristics of the whole group of subjects

*Age (N*=*20)*	
Mean	25.80
Range	20–36

*Years of education (N*=*20)*	
<12 Years	1
⩾12 Years	19

*Monthly household net income (N*=*20)*	
To €1.000	10
To €2.000	7
To €3.000	3

*Frequency of hairwashing per week (N*=*10)*	
Mean	5.5
Range	2–10

*Cosmetic hair treatment (N*=*20)*	
Hair tint	0
Hair coloring	1

*Age at circumcision (N*=*9)*	
On the 8th day of life	2
⩽7th Birthday	3
⩽14th Birthday	1
⩽26th Birthday	3

**Table 2:** Comparisons between circumcised and uncircumcised male individuals

	N	*Mean*	*S.d.*	T	P
*Body mass index*					
(a)	10	25.85	2.85	0.074	0.94
(b)	9	25.76	2.24		

*Subjective stress (PSQ)*					
(a)	11	0.09	0.15	0.788	0.45
(b)	7	0.02	0.21		

*Age*					
(a)	11	26.45	4.55	0.943	0.36
(b)	9	25.00	2.12		

*Physical activity*					
Sports frequency per week					
(a)	9	2.89	1.17	0.502	0.63
(b)	6	2.50	1.64		
Sport intensity					
(a)	10	5.60	2.17	−0.065	0.95
(b)	9	5.67	2.29		

*Anxiety/depressivness (HADS)*					
(a)	11	11.73	6.93	0.206	0.84
(b)	9	11.11	6.43		

*Physical complaints (GBB-24)*					
(a)	11	10.73	7.64	−0.555	0.59
(b)	8	13.25	11.08		
					
*Resilience (RS-13)*					
(a)	11	68.64	9.78	−0.335	0.74
(b)	9	70.22	11.10		

*Sense of coherence (SOC-9L)*					
(a)	11	46.73	4.08	0.816	0.43
(b)	9	44.89	5.67		

Abbreviations: GBB-24, Giessen Complaint List; HADS, Hospital Anxiety and Depression Scale; PSQ, Perceived Stress Questionnaire; RS-13, resilience scale. Two-tailed *t*-test data between (a) uncircumcised male individuals; (b) circumcised male individuals; one GBB-24 and two PSQ questionnaires were not completed.
