# Supplementary material for: Circumcision does not alter long-term glucocorticoids accumulation or psychological effects associated with trauma- and stressor-related disorders
Source: Transl Psychiatry. 2017 Mar 14;7(3):e1063–. doi: 10.1038/tp.2017.23 (PMC5416669; doi:10.1038/tp.2017.23)
Supplement: Supplementary data [file tp201723x1.docx]

| **Supp. Material: Correlations of standardized questionnaires in uncircumcised probands** | | | | | |
| --- | --- | --- | --- | --- | --- |
|  | | Anxiety/Depress-iveness (HADS) | Physical Complaints (GBB-24) | Resilience (RS-13) | Sense of Coherence (SOC9L) |
| Subjective Stress Perception (PSQ) | Pearson’s correlation | .914^**^ | .270 | -.237 | -.590 |
|  | N | 11 | 11 | 11 | 11 |
| Anxiety/Depressiveness (HADS) | Pearson’s correlation | 1 | .172 | -.193 | -.573 |
|  | N | 11 | 11 | 11 | 11 |
| Physical Complaints (GBB-24) | Pearson’s correlation | .172 | 1 | -.625^*^ | -.099 |
|  | N | 11 | 11 | 11 | 11 |
| *Pearson product moment correlation coefficient between HADS,PSQ,GBB-24,RS-13,SOC-9L; *P<.05;**P<.01 (two sided)* | | | | | |

| **Supp. Material: Correlations of standardized questionnaires in circumcised probands** | | | | | |
| --- | --- | --- | --- | --- | --- |
|  | | Anxiety/Depress-iveness (HADS) | Physical Complaints (GBB-24) | Resilience (RS-13) | Sense of Coherence (SOC9L) |
| Subjective Stress Perception (PSQ) | Pearson’s correlation | .901^**^ | .969^**^ | -.789^*^ | -.865^*^ |
|  | N | 7 | 7 | 7 | 7 |
| Anxiety/Depressiveness (HADS) | Pearson’s correlation | 1 | .830^*^ | -.657 | -.854^**^ |
|  | N | 9 | 8 | 9 | 9 |
| Physical Complaints (GBB-24) | Pearson’s correlation | .830^*^ | 1 | -.723^*^ | -.733^*^ |
|  | N | 8 | 8 | 8 | 8 |
| *Pearson product moment correlation coefficient between HADS,PSQ,GBB-24,RS-13,SOC-9L; *P<.05;**P<.01 (two sided); one GBB-24 and two PSQ questionnaires were not completed* | | | | | |
